# Deducing signaling pathways from parallel actions of arsenite and antimonite in human epidermal keratinocytes

**DOI:** 10.1038/s41598-020-59577-0

**Published:** 2020-02-19

**Authors:** Marjorie A. Phillips, Angela Cánovas, Miguel A. Rea, Alma Islas-Trejo, Juan F. Medrano, Blythe Durbin-Johnson, David M. Rocke, Robert H. Rice

**Affiliations:** 1 Department of Environmental Toxicology, University of California, Davis, CA USA; 2 Centre for Genetic Improvement of Livestock, Department of Animal Biosciences, University of Guelph, Guelph, ON N1G 2W1 Canada; 3 Department of Chemistry, Universidad Autónoma Querétaro, Querétaro, Mexico; 4 Department of Animal Science, University of California, Davis, CA USA; 5 Division of Biostatistics, Department of Public Health Sciences, Clinical and Translational Science Center Biostatistics Core, University of California, Davis, CA USA

**Keywords:** Cell biology, Molecular biology

## Abstract

Inorganic arsenic oxides have been identified as carcinogens in several human tissues, including epidermis. Due to the chemical similarity between trivalent inorganic arsenic (arsenite) and antimony (antimonite), we hypothesized that common intracellular targets lead to similarities in cellular responses. Indeed, transcriptional and proteomic profiling revealed remarkable similarities in differentially expressed genes and proteins resulting from exposure of cultured human epidermal keratinocytes to arsenite and antimonite in contrast to comparisons of arsenite with other metal compounds. These data were analyzed to predict upstream regulators and affected signaling pathways following arsenite and antimonite treatments. A majority of the top findings in each category were identical after treatment with either compound. Inspection of the predicted upstream regulators led to previously unsuspected roles for oncostatin M, corticosteroids and ephrins in mediating cellular response. The influence of these predicted mediators was then experimentally verified. Together with predictions of transcription factor effects more generally, the analysis has led to model signaling networks largely accounting for arsenite and antimonite action. The striking parallels between responses to arsenite and antimonite indicate the skin carcinogenic risk of exposure to antimonite merits close scrutiny.

## Introduction

Chronic exposure to inorganic arsenic, primarily in water supplies, has numerous deleterious effects on humans, including cancer at several anatomic sites^1^. Many mechanisms have been proposed for these effects, indicating possible interference of arsenic with a variety of signaling pathways^2^ to which epigenetic changes may contribute^3^. Proteins with vicinal thiols, including zinc finger DNA repair proteins^4^, are particularly attractive as potential targets with deleterious downstream effects. Continuing exposure of large populations has evoked considerable effort to elucidate the broad consequences for health and the mechanisms that lead to their manifestation.

Also of concern, including human therapeutic treatment for leishmaniasis^5^, considerable exposure occurs to inorganic antimony, a metalloid immediately below arsenic in the periodic table to which it exhibits chemical similarity^6^. The presence of antimony is increasing in the environment through use in small arms ammunition, as a catalyst in plastic, as a flame retardant, and through watershed pollution by mining waste or by recycling operations. This has raised concern for public health, sustainable agriculture and ecosystem effects^7,8^. Inasmuch as antimony trioxide is a rodent carcinogen^9^, finding commonalities in actions of antimonite (SbIII) and arsenite (AsIII) would assist in understanding their mechanisms of action *in vivo*.

Human epidermis is a known target for carcinogenic effects of arsenic. Cultured human epidermal keratinocytes, a model system for studies of effects of chemicals on epidermis, have been shown to respond similarly to treatment with arsenite and antimonite. As shown using the normal cells and a minimally deviated line of spontaneously immortalized human epidermal keratinocytes (SIK), both metalloids suppress many aspects of the cell differentiation program while preserving the proliferative potential of the cells^10–13^. Recently, protein and transcriptional profiling experiments using SIK demonstrated that changes due to treatment with these two agents occur in parallel^14^. The initial targets and mechanisms of action, and how similar these are for arsenite and antimonite, remain to be determined. For this purpose, we have analyzed profiling data to predict cell signaling pathways and transcription factors that appear to mediate downstream effects. Global analyses of changes in proteins and mRNAs now permit a more general approach to the identification of signaling pathways and transcription factors that drive the responses. As a result, three previously unsuspected signaling pathways for these metalloids have been revealed and validated in keratinocytes, and model signaling networks largely accounting for arsenite and antimonite action are presented.

## Results

### Parallel changes in transcript and protein levels

**Figure 1:**
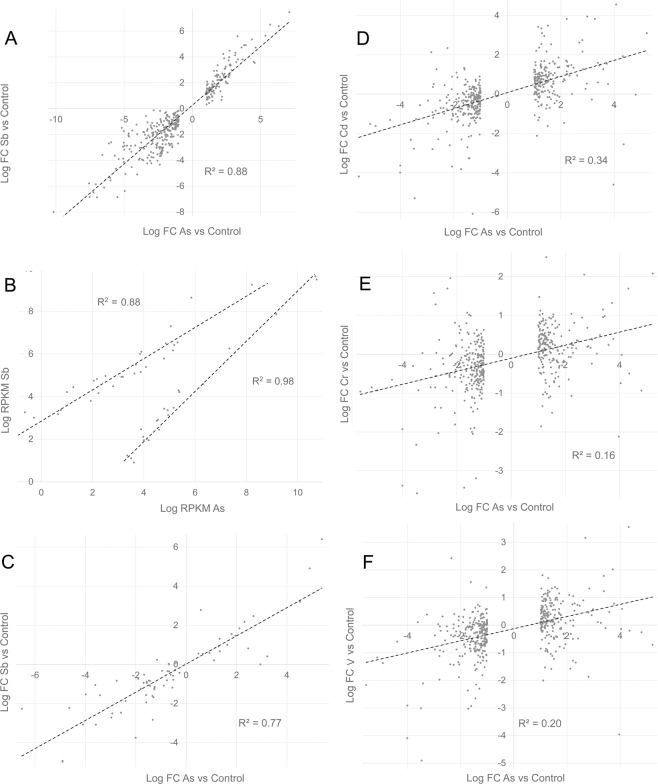
Comparison of effects of treatments on differentially expressed genes and proteins. Log fold change of genes (**A**) or proteins (**C**) differentially expressed by arsenite compared to control were plotted against log fold change of the same genes or proteins after antimonite treatment compared to control. Panel B shows genes with significantly different responses to arsenite and antimonite plotted as log RPKM. The upper line shows genes more responsive to antimonite, and the lower line shows genes more responsive to arsenite. Similarly, log fold changes of genes differentially expressed by arsenic compared to control were plotted against log fold changes of the same genes after treatments with cadmium chloride (**D**), potassium chromate (**E**) or sodium orthovanadate (**F**). Data were derived from next generation sequencing (**A**–**C**) or DNA microarray (**D**–**F**).

Applying the parameters p < 0.05 and fold change >2, 387 and 598 differentially expressed mRNAs were identified between untreated (control) and arsenite or antimonite treatments, respectively, while the number of differentially expressed mRNAs comparing samples from arsenite- and antimonite-treated cells was 49, an order of magnitude fewer. This relationship, consistent with our previous report^14^, can be visualized as a scatterplot of log fold change of genes differentially expressed after arsenite treatment vs. log fold change of those genes after antimonite treatment (Fig. 1A). Not previously recognized, the present analysis reveals that the 49 genes differing in their transcriptional responses to arsenite and antimonite also show a strong concordance of response. The basis of their difference was sensitivity; approximately half were more responsive to arsenite and the other half were more responsive to antimonite (Fig. 1B). A scatterplot of differentially expressed proteins identified in our earlier study also shows a high level of concordance of responses to arsenite and antimonite (Fig. 1C).

Emphasizing the remarkable concordance of response between arsenite and antimonite, transcriptional responses to arsenite were not parallel with responses to several metal compounds. Although arsenate and arsenite responses were concordant in our DNA microarray studies^15^, such studies showed poor concordance in response to arsenic treatment compared to cadmium, chromate or vanadate (Fig. 1D–F), not previously reported.

### Pathway analysis

Using the lists of differentially expressed genes identified here and proteins identified in our earlier study, Ingenuity Pathway Analysis (IPA) software was used to explore signaling pathways and upstream regulators resulting from arsenite and antimonite exposure. Results indicate that arsenite and antimonite produce similar effects in keratinocytes because they target many of the same metabolic pathways and regulators, leading to significant overlap in differentially expressed genes and proteins.

#### Canonical pathways

Based on analysis of differentially expressed genes, Table 1A shows the merged five top pathways enriched for genes affected by arsenite or antimonite treatment and the numbers of genes on which the predictions are based. Four of these predicted pathways are identical (Nrf2-mediated oxidative stress, glucocortcoid receptor signaling, xenobiotic metabolism signaling, γ-glutamyl cycle), while vitamin C transport is in the top five only for antimonite treatment, and retinoate biosynthesis is affected only by arsenite treatment. As seen in the full list of genes employed in the analysis (Supplementary Table S1), many of the same genes (76%) are included on lists of both arsenite-affected and antimonite-affected genes. The genes responsible for effects on Nrf2-mediated oxidative stress response, xenobiotic metabolism signaling, vitamin C transport and γ-glutamyl cycle pathways overlap substantially with each other, likely all a result of Nrf2 (*NFE2L2*) activation. Proteomic analysis also predicted the involvement of the Nrf2-mediated oxidative stress response as a top affected pathway (Table 1B).

**Table 1:** Canonical pathways enriched for genes affected by arsenite and antimonite.

Pathway	p-value As	p-value Sb	Overlap As	Overlap Sb
**A. Transcriptional Analysis**				
Nrf2-mediated Oxidative Stress Response	2.54E-10	3.76E-11	21/193 10.9%	27/193 14%
Glucocorticoid Receptor Signaling	4.69E-05	1.60E-05	19/339 5.6%	26/339 7.7%
Retinoate Biosynthesis I	4.71E-05	not listed	6/34 17.6%	(4/34 11.7%)
Xenobiotic Metabolism Signaling	6.74E-05	2.33E-06	17/290 5.9%	25/290 8.6%
γ-Glutamyl Cycle	1.26E-04	4.04E-05	4/14 28.6%	5/14 35.7%
Vitamin C Transport	(1.64E-04)	8.38E-05	(4/16 25.0%)	5/16 31.2%
**B. Proteomic Analysis**				
Nrf2-mediated Oxidative Stress Response	4.24E-06	1.34E-04	8/193 4.1%	7/193 3.6%
Pentose Phosphate Pathway	1.26E-05	2.21E-05	3/10 30%	3/10 30%
Caveolar-mediated Endocytosis Signaling	2.34E-05	(8.13E-03)	5/71 7%	(3/71 4.2%)
Integrin Signaling	9.05E-05	(8.38E-02)	7/219 3.2%	(4/219 1.8%)
Pentose Phosphate Pathway (Non-oxidative)	3.38E-04	(4.91E-04)	2/6 33.3%	(2/6 33.3%)
Methylglyoxal Degradation III	(2.62E-03)	1.00E-04	(2/16 12.5%)	3/16 18.8%
Aldosterone Signaling in Epithelial Cells	(8.44E-03)	5.20E-05	(4/166 2.4%)	7/166 4.2%
Glutathione Biosynthesis	(1.43E-02)	9.93E-05	(1/3 33.3%)	2/3 66.7%

Summary of the merged top five canonical pathways predicted by IPA to be affected by arsenite (As) and antimonite (Sb) treatments, sorted by p-values for analysis of untreated vs. arsenite-treated samples, which are shown in the first column. The second column shows p-values for analysis of untreated vs. antimonite-treated samples. The top five predicted pathways did not overlap completely for arsenite and antimonite treated samples. When a canonical pathway was predicted to be affected, but was not in the list of the top five, p-values are shown in parentheses. The third and fourth columns show the overlaps of differentially expressed genes or proteins with the molecules in the pathway and as a percentage. Part A are predictions based on transcriptional analysis of differentially expressed genes and Part B are predictions based on proteomic analysis.

#### Predicted upstream regulators

The list of top five regulators, predicted by analysis of differentially expressed genes due to arsenite and antimonite treatments, overlap for four of the top five predicted molecules (Table 2A). Two of these are expected as upstream regulators of arsenite effects: arsenite itself and *NFE2L2* (Nrf2), a transcription factor induced by arsenite treatment, and both are predicted to be activated. The predicted activation of these two regulators after antimonite treatment also provides strong support for the hypothesis that arsenite and antimonite overlap in their mechanisms.

**Table 2:** Predicted upstream regulators.

Regulator	p-value As	p-value Sb	Prediction
**A. Transcriptional Analysis**			
OSM	1.56E-24	5.72E-23	Activated by Sb
Dexamethasone	7.18E-23	7.99E-26	No prediction
NFE2L2	2.73E-21	6.96E-21	Activated by As and Sb
TNFα	1.78E-17	3.83E-21	Activated by Sb
Arsenic trioxide	1.57E-16	(1.70E-11)	Activated by As and Sb
MYC	(1.18E-13)	9.57E-18	No prediction
**B. Proteomic Analysis**			
MYC	2.83E-16	3.44E-19	No prediction
ROCK2	5.04E-16	3.47E-15	Inhibited by As and Sb
FOS	1.09E-14	(4.40E-11)	No prediction
JUN	3.35E-13	(7.96E-10)	No prediction
EFNA4	3.38E-13	5.84E-16	Inhibited by As and Sb
17-Beta-estradiol	(1.09E-12)	9.14E-16	No prediction
EFNA3	(2.74E-11)	4.86E-14	Inhibited by As and Sb

Summary of IPA predicted upstream regulators based on differentially expressed genes (A) or proteins (B). The top five predicted upstream regulators are sorted by p-value for changes due to arsenite treatment (As), which are shown merged in the first column. The second column shows p-values for analysis of untreated vs. antimonite-treated samples (Sb). The top five predicted upstream regulators did not overlap completely for arsenite and antimonite treated samples. When a regulator was predicted, but was not in the list of the top five, p-values are shown in parentheses. The last column indicates whether the regulator is predicted to be activated or inhibited or whether no prediction was made.

The identification of dexamethasone as an upstream regulator in keratinocytes is consistent with well-known clinical effects of corticosteroids in dermatology and with the identification of glucocorticoid signaling as an enriched canonical pathway. The identification of oncostatin M (OSM) and tumor necrosis factor alpha (TNFα) may help to explain keratinocyte-specific effects since these cytokines are known to target this cell type^16,17^. Sorted by z score (activation score), 45 and 85 regulators are predicted to be activated by arsenite and antimonite, respectively (24 in common), and 33 and 30 predicted to be inhibited (11 in common) (Supplementary Tables S2 and S3). Top shared inhibited regulators are NRG1 (an EGF receptor family ligand), and isotretinoin (13-cis retinoic acid), both of which have complex effects on keratinocyte proliferation and differentiation^18–20^. Other predicted inhibited regulators were calcium ion, EFNA1 and FGF10, all reported to increase expression of several markers of keratinocyte differentiation^21–23^. The transcription factor DDIT3 and signaling molecules KRAS and STAT4 are also predicted to be activated by both agents.

The predicted activities of upstream regulators show remarkable similarity for arsenite and antimonite treated samples, as displayed in a heat map of top regulators (sorted by p value) (Supplementary Fig. S1A). Filtering the results for genes, proteins and RNAs to eliminate exogenous chemicals (Supplementary Fig. S1B) showed that the main difference is TNFα (predicted to be activated by antimonite, but not arsenite).

The list of top five upstream regulators predicted by proteomic analysis (Table 2B) differs from those predicted by transcriptional analysis, although most appear as lower-ranked entries on the latter. Three of these top regulators appear on both arsenite and antimonite lists: Myc, EFNA4 and ROCK2. All of these have demonstrated effects on keratinocyte differentiation and proliferative potential. Sorted by z score, five and seventeen regulators were predicted to be activated by arsenite and antimonite, respectively (three in common: NFE2L2, GLI2 and 1,2- dithiol-3-thione, an activator of NFE2L2) and thirteen and sixteen, respectively, were predicted to be inhibited (eight in common: ROCK2, GLIS1, EFNA1, 2, 3,and 4, TNFSF11 and isotretinoin).

Overall, these predicted upstream regulators from analysis of arsenite and antimonite treated samples display extensive overlap and suggest potential shared targets for exploration to explain downstream effects. The impact of arsenite on NFE2L2 and its consequences have been well documented in many cell types^24^. Identification of pathways and upstream regulators with known effects on keratinocyte differentiation and proliferative potential are intriguing. In particular, ROCK inhibitors have been shown to impact proliferative potential of keratinocytes^25^ and may contribute to the observed maintenance of colony forming efficiency by arsenite and antimonite^11^. Both arsenite and antimonite also suppress keratinocyte differentiation^10–13^, which could be due to effects on many of these upstream regulators with demonstrated roles in this process. Among these are Myc, the ephrin signaling system (including the ligand family, EFNA, and receptor families, EPHA and EPHB), GLI2, retinoic acid receptors and ligands (including isotretinoin), corticosteroids (including dexamethasone) and OSM.

### Experimental evidence for oncostatin M as an upstream regulator

Since OSM is the top predicted upstream regulator of transcriptional changes induced by arsenite and antimonite, experiments were designed to determine whether treatment of cells with OSM would elicit responses similar to those observed after treatment with arsenite and antimonite, especially effects on differentiation and proliferative potential. Because we have been investigating effects of chronic treatment (days rather than hours) with arsenite and antimonite, we used similar treatment times for our experiments with OSM, with treatments starting at confluence, just as the cells begin differentiating.

**Figure 2:**
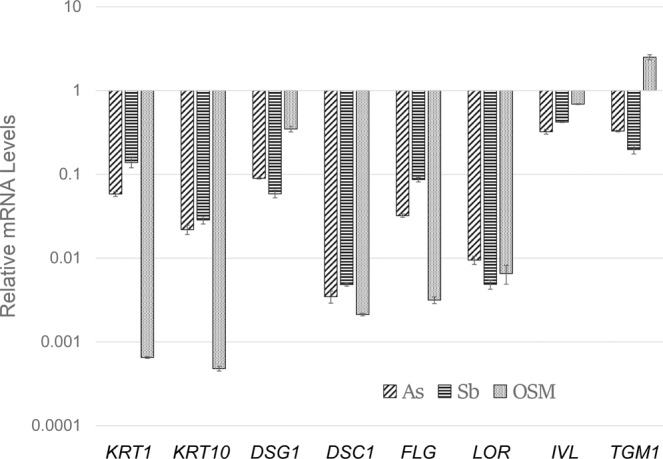
Suppression of differentiation marker expression by arsenite (As), antimonite (Sb) and oncostatin M (OSM). Relative mRNA amounts were determined by qPCR and normalized to amounts in untreated samples, set at 1. Results are the averages and ranges of two samples. Most treated samples are significantly different from the untreated control at p ≤ 0.001; IVL treated with OSM and TGM1 treated with Sb have p values of 0.035.

**Figure 3:**
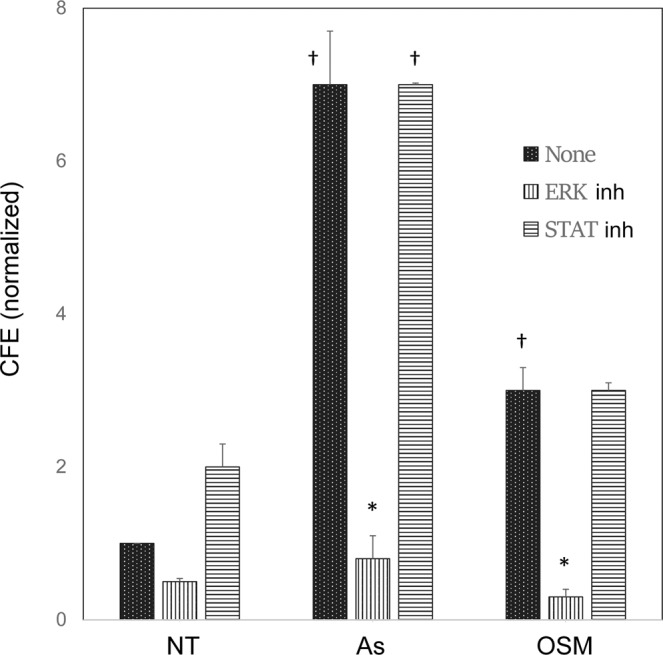
Stimulation of colony formation by arsenite and oncostatin M (OSM). Values for colony forming efficiency (CFE) from triplicate cultures are given relative to no treatment (NT) or treatment with 2 µM arsenite (As) or 50 ng/ml OSM. Effects of inhibitors of the Erk (U1026, 10 µM) and Stat (Jak Kinase inhibitor 1, 1 µM) pathways are indicated (ERK inh, STAT inh). A representative result is shown. In a compilation of 2–6 experiments with various combinations of conditions, stimulation of CFE compared to NT was highly significant for As and OSM (^†^p < 0.02) and inhibition of CFE by the Erk inhibitor (*p < 0.02).

Like arsenite and antimonite, OSM treatment had a large inhibitory effect on induction of a subset of differentiation markers: KRT1, KRT10, DSG1, DSC1, LOR and FLG mRNAs (Fig. 2). Although arsenite and antimonite have substantial effects on these mRNAs, OSM is a better suppressor than either arsenite or antimonite for several of these. In contrast to arsenite and antimonite, OSM had only a modest effect on levels of IVL mRNA, while increasing TGM1 mRNA. Finally, like arsenite and antimonite^11^, chronic OSM treatment increased the proliferative potential of cultured keratinocytes after confluence compared to untreated cultures as measured by colony forming efficiency (Fig. 3, dark bars).

**Figure 4:**
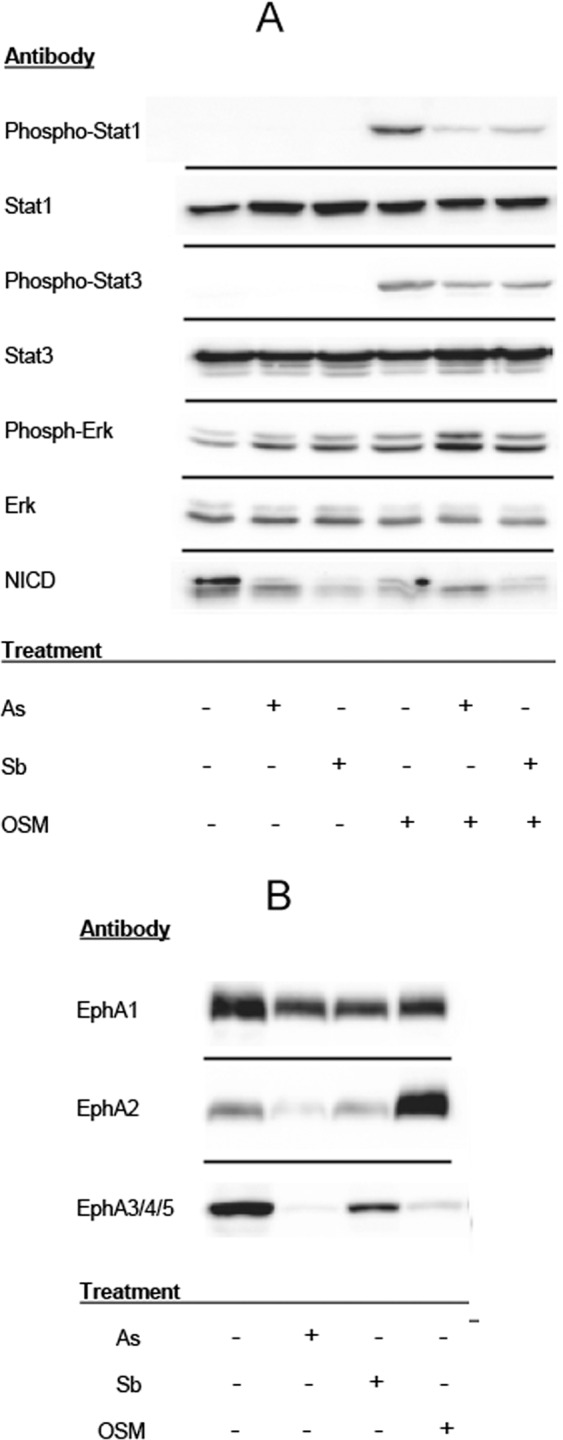
Analysis of treatment effects by immunoblotting. (**A**) Effects of arsenite (As), antimonite (Sb) and OSM on signaling pathways. SIK cultures were treated at confluence for 7 days with 3 µM arsenite, 6 µM antimonite, 50 ng/ml OSM or combinations as indicated, then harvested for immunoblotting with the indicated antibodies. (**B**) Alteration of ephrin receptor levels by arsenite (As), antimonite (Sb) and OSM. SIK cultures were treated at confluence for 6 days with 3 µM arsenite, 6 µM antimonite or 50 ng/ml OSM, then harvested for immunoblotting. For panel A, the same set of treated cell extracts was run on parallel gels, blotted and probed with the indicated antibodies. For panel B, a different set of cell extracts was run on parallel gels, blotted and blotted probed for the ephrins. Original images of the blots and molecular weight markers are shown in Supplementary Figure 4 from which the relevant areas of interest were cropped (separated by horizontal lines above).

OSM has been demonstrated to act through Stat signaling (Stats 1 and 3) and Erk signaling pathways (reviewed by^26^). Chronic exposure of keratinocytes to OSM increased phospho-Stat1, -Stat3, and -Erk, as expected (Fig. 4A). Arsenite or antimonite treatments alone had no effect on Stat1 or Stat3 phosphorylation while they stimulated Erk phosphorylation. Simultaneous treatment with OSM and arsenite or antimonite decreased Stat1 phosphorylation compared to OSM alone and had little impact on OSM-induced Stat3 or Erk phosphorylation.

This result raises the question of which signaling pathways are required for suppression of differentiation marker expression and increase in proliferative potential by arsenite, antimonite and OSM. Inhibition of the Jak kinases, responsible for Stat phosphorylation, was partially able to reverse decreases in differentiation markers by OSM. Inhibition of the kinase that phosphorylates Erk had only a small effect, but the combination of the two inhibitors completely blocked OSM effects. In contrast, only an inhibitor of Erk phosphorylation affected the response to arsenite and antimonite (Supplementary Table S4). A plausible reason why OSM had a larger impact than arsenite and antimonite on a subset of differentiation markers might be due to independent, negative effects of both Stat and Erk signaling pathways on expression of the affected genes. OSM signals through both Stat and Erk pathways, while arsenite and antimonite signal only through Erk. In contrast to inhibitor effects on differentiation markers, only an Erk pathway inhibitor blocked the increased proliferative potential elicited by both arsenite and OSM (Fig. 3, vertically vs horizontally striped bars).

### Impact of corticosteroid signaling on responses to arsenite and antimonite

**Figure 5:**
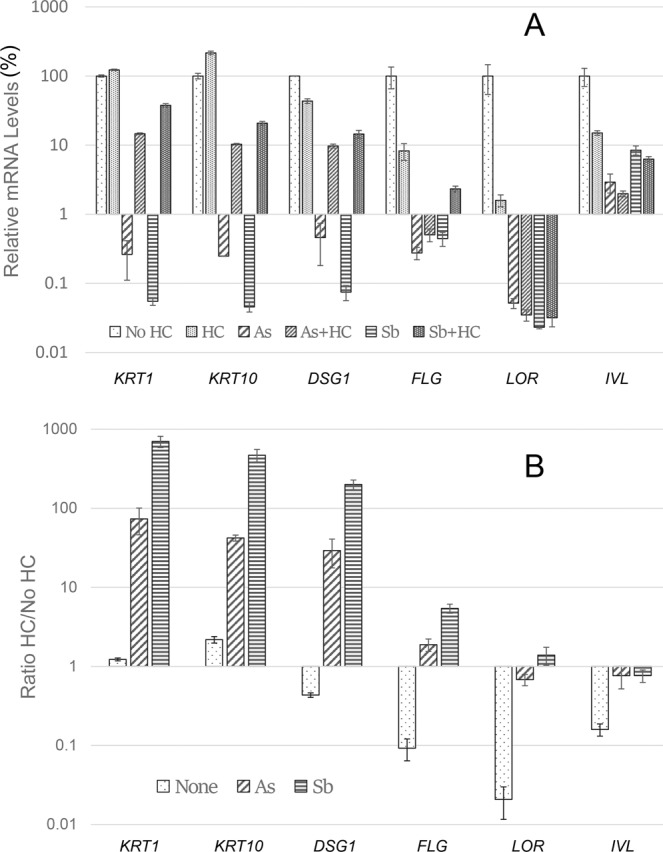
Effect of hydrocortisone (HC) on transcriptional responses to arsenite (As) and antimonite (Sb) treatments. (**A**) Relative mRNA amounts were determined by qPCR and normalized to amounts in samples without HC, set at 100%. Results are the means and standard deviations of 3 samples. (**B**) Ratios were calculated for treatments in the presence of hydrocortisone compared to those in the absence of hydrocortisone. Hydrocortisone significantly altered the response at p ≤ 0.05 except for the following: FLG in samples treated with As, and IVL in samples treated with As and Sb.

The prediction of dexamethasone as an upstream regulator suggests that responses to arsenite and antimonite may be different in the presence and absence of corticosteroids. Under standard culture conditions, arsenic oxides have been shown to suppress various markers of differentiation^27^. Responses to antimonite are similar (Fig. 5A). Present work demonstrates that suppression of several differentiation markers by both arsenite and antimonite was diminished by addition of hydrocortisone, the major steroid in the culture medium. The differences in the ratios of suppression in the presence and absence of hydrocortisone ranged from several hundred fold down to two fold (Fig. 5B). Most dramatically affected were *KRT1*, *KRT10* and *DSG1*. For these genes, hydrocortisone alone did not greatly affect amounts of mRNAs, but instead diminished the responses to arsenite and antimonite. For another set of genes (*FLG*, *LOR* and *IVL*), the fold suppression by arsenite and antimonite was altered, but this was predominantly due to changes induced by hydrocortisone alone. The suppressive effects of corticosteroids on these genes have also been observed *in vivo*^28^. For all of these genes, hydrocortisone affected the responses to arsenite and antimonite similarly. Responses to dexamethasone were similar to those obtained with hydrocortisone.

### Impact of arsenite and antimonite on ephrins, predicted upstream regulators

Since ephrins and their receptors have significant impacts on keratinocyte differentiation (reviewed by^29^) and were identified by IPA as upstream regulators after arsenite and antimonite treatment, we examined effects of arsenite and antimonite on levels of ephrin and ephrin receptor mRNA expression. Seven ephrin mRNAs and seven ephrin receptor mRNAs were expressed at moderate levels (>1 RPKM). Three ephrin receptor mRNAs (EPHA1, EPHA2 and EPHA4) and one ephrin mRNA (EFNB2) were decreased two fold or more by arsenite. These decreased receptor mRNAs led to decreases in the encoded proteins by both arsenite and antimonite as demonstrated by Western blotting (Fig. 4B). OSM, which also had suppressive effects on a subset of the differentiation markers affected by arsenite and antimonite, decreased EphA1 and EphA4 while increasing EphA2. Effects of OSM on EphA2 have been previously reported^30^. The number of different ephrin receptors and their abilities to bind many or all of the ligands in their class (A or B) makes ascribing particular functions to individual receptors difficult. While responses to particular ephrins are largely overlapping, some differences have been demonstrated^22^, suggesting that receptors distinguish among the ligands and initiate different downstream signaling. Perhaps EphA2 is of particular importance for regulation of *TGM1*, which was suppressed by arsenite and antimonite, but increased by OSM, while regulation of *KRT1*, *KRT10*, *DSG1* and *DSC1*, which were decreased by all three agents, may be under the control of EphA1 and/or EphA4. All of these markers have been shown to be in the set of ephrin up-regulated genes^22^.

### Predicted regulation of transcription factors by arsenite and antimonite

The IPA prediction of upstream regulators that might be affected by arsenite and antimonite relies on analysis of the patterns of gene expression observed after treatment. An alternative approach to explain some of the transcriptional effects of these treatments is to identify transcription factors that are themselves altered by treatment (regardless whether they are primary targets). To this end, we filtered the gene expression data for transcription factors that were differentially expressed at the statistical significance of p ≤ 0.05. Supplementary Table S5 presents the transcription factors altered by arsenite treatment. Of the 30 factors identified, 24 are also altered by antimonite at a similar level of statistical significance. Twelve of these 30 transcription factors have been demonstrated to affect keratinocyte differentiation (IRF6, EHF, TP63, KLF5, CEBPA, BARX2, FOXQ1, HOPX, FOS, GRHL1, ELF3 and PITX1) while nine have been shown to affect proliferative potential of cells (IRF6, EHF, BTG2, TP63, KLF5, CEBPA, CDKN2B, HOPX and ELF3)^31–51^.

**Figure 6:**
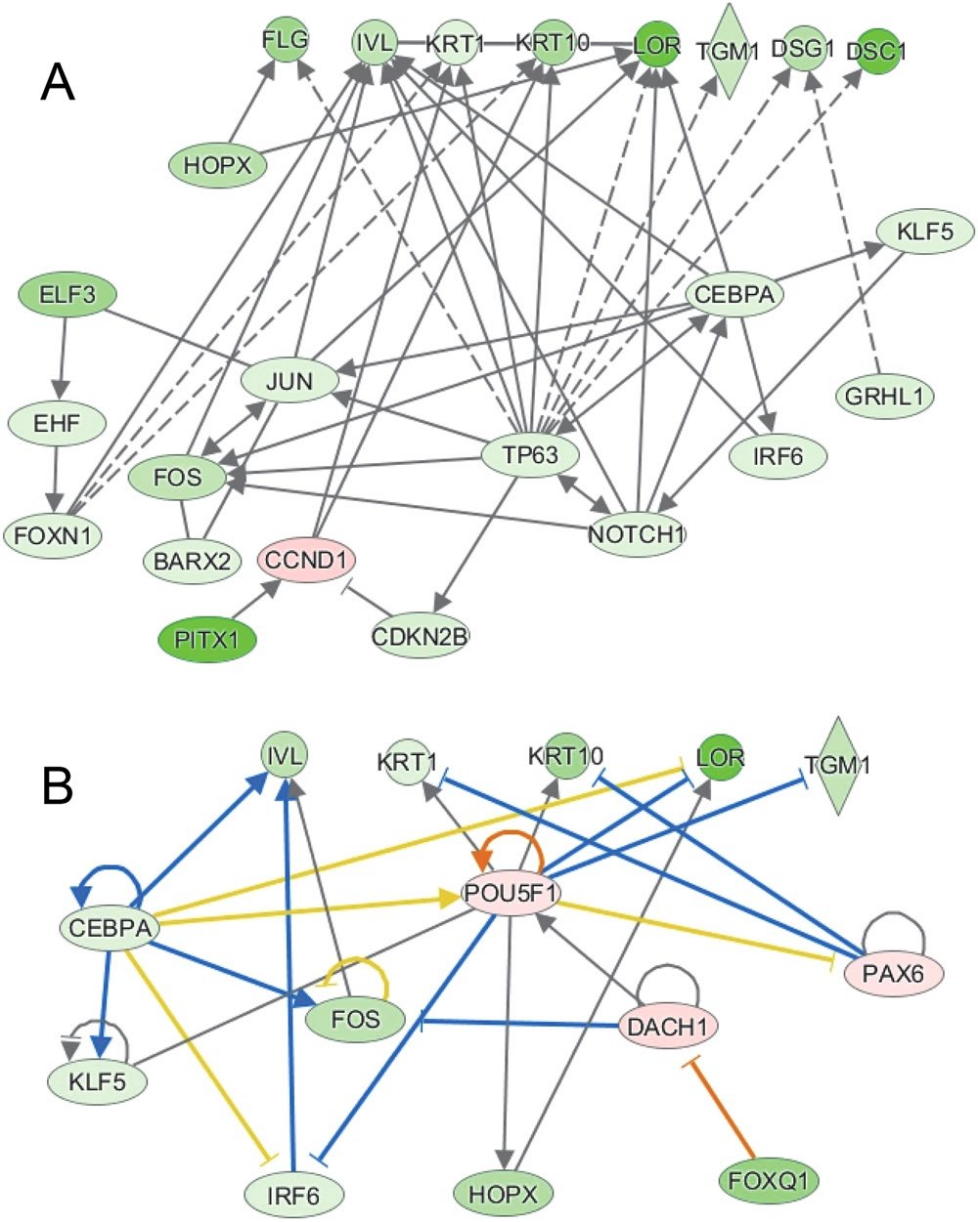
IPA generated networks of transcription factors and differentiation markers. (**A**,**B**) Custom IPA networks were generated from selected lists of keratinocyte differentiation markers and transcription factors shown by transcriptional analysis to be altered by arsenite and antimonite treatments. Molecules in green were decreased in the datasets with darker shades indicating more suppression. Molecules in pink were increased in the dataset. Blue lines are predicted to lead to inhibition and orange lines to activation. Yellow lines indicate findings opposite to predictions, while black lines indicate no prediction of activation or suppression. The networks were generated through the use of Ingenuity Pathway Analysis version 2018 (QIAGEN Inc., https://www.qiagenbioinformatics.com/products/ingenuity-pathway-analysis)^62^.

A network of this first group of transcription factors, demonstrating predicted regulation of several downstream differentiation markers, is shown in Fig. 6A. Also included are NOTCH1 and FOXN1, shown previously to regulate keratinocyte differentiation. Major hubs occur with TP63, CEPBA, NOTCH1, FOXN1 and JUN/FOS. Most of the transcription factors connect directly to the differentiation marker genes or connect through one intermediate transcription factor. These are not all of the possible connections, but represent the most direct paths between transcription factors and their targets. A separate network illustrating possible regulation of differentiation marker expression by FOXQ1 is shown in Fig. 6B. The connection of FOXQ1 to downstream targets is less direct, involving more intermediates. These two networks, based on evidence gleaned from the literature, support the hypothesis that these transcription factors, shown to be altered by arsenite and antimonite treatment, could be responsible, for much of the down-regulation of differentiation markers by these agents.

## Discussion

Examination of affected canonical pathways revealed that many of the same differentially expressed genes led to the predicted perturbation of the top pathways, suggesting connections among these pathways. Among several compounds that induce Nrf2-mediated oxidative stress response in keratinocytes, judging by their abilities to induce HO1, only arsenite and antimonite induce a robust and sustained response^15^. Perhaps this sustained response helps rationalize their unique abilities to suppress keratinocyte differentiation while maintaining proliferative potential. However, hyperactivation of a single signaling pathway could produce a contrasting result. For example, unopposed activity of the Nrf2-mediated oxidative stress response by KEAP1 ablation leads to epidermal hyperkeratosis and increased expression of keratinocyte differentiation markers in mice^52^.

The lists of top upstream regulators predicted by transcriptional and proteomic analysis are intriguing and provide some validation of the predictive process. That arsenic trioxide is predicted to be activated by both arsenite and antimonite is especially supportive. Most of the other regulators have already been identified as important regulators of keratinocyte differentiation and proliferative potential. OSM, ephrins (e.g. EFNA3 and EFNA4) and dexamethasone all have been demonstrated to affect transcription of differentiation markers^22,28,30^, while MYC has been shown to drive cells from the stem cell compartment and initiate the differentiation process^53^. FOS and JUN have been identified as transcription factors important for expression of several keratinocyte differentiation markers, reviewed in^54^. Finally ROCK inhibitors prolong the proliferative capacity of cultured keratinocytes^25^. The predicted inhibition of ROCK2 by arsenite and antimonite could rationalize their abilities to maintain proliferative potential.

Three of the predicted upstream regulators have been experimentally validated. First, OSM, predicted to be activated by antimonite, did indeed demonstrate many similarities to arsenite and antimonite. All three agents effectively suppressed a subset of differentiation markers while maintaining proliferative potential. Second, corticosteroid agonists such as dexamethasone and the endogenous agonist hydrocortisone acted similarly to arsenite and antimonite by suppressing FLG, LOR and IVL expression (effects on FLG and LOR previously reported by^28^) while antagonizing arsenite and antimonite suppression of several other differentiation markers. These mixed responses are consistent with a z-score that predicted neither activation nor inhibition. Finally, ephrins and their receptors have been shown to promote keratinocyte differentiation^22^ and were predicted upstream regulators of arsenite and antimonite downstream effects. We have shown here that amounts of several ephrin receptors were decreased by these metalloids.

## Conclusions

Based on transcriptional and proteomic comparisons and Ingenuity pathway analysis, showing that keratinocytes respond nearly identically, we conclude that arsenite and antimonite share virtually the same signaling pathways. Evidently, three unanticipated upstream regulators, verified here experimentally, participate in the response. In addition, several transcription factors appear affected by arsenite and antimonite, and a model network showing how these affect differentiation markers has been constructed. A goal of future efforts will be to connect affected transcription factors with signaling pathways and ultimately with direct targets of arsenite and antimonite. Thus, present results emphasize the likelihood arsenite and antimonite act in parallel as human skin carcinogens.

## Materials and Methods

### Sample preparation for transcriptomic and proteomic analyses

These experiments used a line of spontaneously immortalized epidermal keratinocytes (SIK)^55^. The cells were cultured as previously described, under which conditions normal and neoplastic human keratinocyte lines exhibit highly similar responses to arsenic treatment, and normal human keratinocytes give qualitatively the same patterns of gene expression at different concentrations of arsenic and different time points^15^. Cultures were treated with similarly effective concentrations of sodium arsenite (3 µM) and potassium antimony tartrate (6 µM), as shown previously for transcriptional responses and confirmed in this work for survival (Supplementary Fig. S2). Near the maximally tolerated concentrations for the 7 day exposure period, while approximating exposures of ≈200 ppb commonly found in well water in geographical regions at risk^56^, this treatment maximized the transcriptional responses. Sample preparation, library synthesis (using a TruSeq RNA Sample Prep Kit from Illumina, Inc), transcriptomic analysis using RNA-Sequencing technology and proteomic analyses were performed as described^14^; to permit accurate signaling pathways analysis, two additional experiments were processed to add to the two previously analyzed. Thus a total of 12 samples, four each from untreated control (n = 4), arsenite-treated (n = 4) and antimonite-treated (n = 4) human epidermal keratinocyte cultures, were analyzed by RNA-sequencing. Each of the four pairs of arsenite and antimonite-treated samples, along with the untreated control, was derived from a separate experiment performed at different times. An average of 35 million sequence reads was obtained for each individual sample. These were assembled and mapped to the annotated human genome assembly (release 80). In all samples, ~90% (88–91%) of the reads were mapped to the human reference sequence. Differential gene expression analysis between untreated (control) and treated (arsenite or antimonite) human epidermal keratinocytes was performed by *t*-testing with correction for false discovery rate.

For access to original transcriptional data, the sequencing results (4 samples each of untreated, As-treated and Sb-treated cultures) have been deposited into the NCBI SRA repository (Temporary Submission ID: SUB4618061) with accession #PRJNA510150. For access to the original proteomic data, the raw data were deposited in the Massive Proteomics repository (massive.ucsd.edu ID #MSV000082992) and ProteomeExchange http://proteomecentral.proteomexchange.org (ID # PXD011259).

### Ingenuity pathway analysis

Transcriptomic datasets comparing arsenite or antimonite treated samples to untreated samples were uploaded into the Qiagen Ingenuity Pathway Analysis program. Core analysis was performed, filtering for fold change ≥2, expression intensity (RPKM) ≥0.2 and false discovery rate ≤ 0.2.

### DNA microarray

SIK cultures were treated starting just before confluence and harvested for analysis after 10 days of treatment with 1 µM sodium arsenite, 3 µM sodium arsenate, 10 µM cadmium chloride, 5 µM potassium chromate or 10 µM sodium vanadate, maximally tolerated concentrations that produced equivalent suppression of differentiation (involucrin mRNA level). cDNA was synthesized using total RNA with oligo dT priming followed by second strand RNA synthesis using biotin-labeled nucleotides as previously described^15^. Fragmented cRNA was hybridized to U-133A arrays (Affymetrix, Santa Clara, CA), and the hybridization was detected with streptavidin-labeled phycoerythrin. After measurement of hybridization intensities, the.CEL files were imported into an R language environment using Bioconductor software and subjected to quantile normalization. An arithmetic average of 16 normalized intensity values from PM probes were transformed using the general logarithm transformation (glog)^57^. The processed data are available in the Dryad Digital Repository (10.5061/dryad.t4q2nc8). Prior to analysis, probes with present calls in fewer than 2 samples were filtered, leaving 11,952 probes. Differential expression analyses were conducted using the Bioconductor package limma, version 3.38.3^58^, with expression modeled by group using a single factor linear model. The analysis incorporated variance weights for log expression calculated using the function vooma within limma^59^. Analyses were conducted using R, version 3.5.3 (R Core Team, 2019). Each treatment was analyzed in two independent cultures. The duplicates of arsenic consisted of one arsenite and one arsenate culture, previously shown to be equivalent in transcription^15^. A multi-dimensional scaling plot of the results showed that the arsenic samples were well separated from the cadmium, chromate and vanadate samples, but the last two were not clearly separated from each other (Supplementary Fig. S3). The number of differentially expressed probes (with false discovery rate adjusted p < 0.05) between each treatment and control was 1036 (arsenic), 808 (cadmium), 184 (chromate) and 522 (vanadate).

### Real time PCR

SIK cultures were treated at confluence with 50 ng/ml Oncostatin M (OSM) (R&D Systems), 3 µM sodium arsenite, 6 µM potassium antimony tartrate (antimonite) or not treated. In some experiments cell cultures were pretreated for 30 min with 10 µM U1026 (an inhibitor of MEK1 and MEK2) (LC Laboratories) or 1 µM JAK Inhibitor I (Calbiochem) before addition of OSM, arsenite or antimonite. To test the influence of hydrocortisone on arsenite or antimonite effects, SIK cultures were grown to confluence in normal culture medium, then switched to medium containing serum depleted of steroids by charcoal/dextran treatment^60^ in the presence and absence of combinations of 10 µM hydrocortisone, 3 µM arsenite and 6 µM antimonite. RNA was prepared using Trizol reagent (Life Technologies), followed by cDNA preparation using an Applied Biosystems reverse transcription kit. cDNA was analyzed by qPCR with Taqman assays (Applied Biosystems), normalizing to MAPK1, a gene product demonstrated by transcriptomic analysis not to be changed by arsenite and antimonite treatments.

### Immunoblotting

Immunoblotting was performed as described^2^ with antibodies obtained from Cell Signaling Technologies: EphA1 (rabbit monoclonal D6V71), EphA2 (rabbit monoclonal D4A2), EphA3/A4/A5 (rabbit monoclonal D2C11), Erk1/2 (rabbit monoclonal 137F5), phospho-Erk1/2 (rabbit monoclonal 20G11), Stat1 (rabbit monoclonal 43H3), phospho-Stat1 (rabbit monoclonal D4A7), Stat3 (rabbit monoclonal D3Z2G), phospho-Stat3 (rabbit monoclonal D3A7) and cleaved Notch1 (rabbit monoclonal D3B8). Antibodies were used at a 1:1000 dilution of the supplied stock following the protocols specified by the supplier. Blots were developed using Thermo Fisher ECL Plus and visualized with a My ECL imager. Image acquisition time was adjusted to visualize all bands without saturating the brightest bands (from 30 seconds to 30 minutes depending on the efficacy of the antibody). Data were analyzed using Thermo My Image Analysis software, version 2.0. Images were inverted to display dark bands on a light background and in some cases brightness or contrast were adjusted to show lighter bands more distinctly, again without saturating the darkest bands. Images were then cropped, arranged and converted to TIFs with Photoshop, version CS6.

### Colony forming efficiency

Confluent SIK cultures were untreated or treated with 50 ng/ml OSM or 3 µM arsenite in the presence and absence of 10 µM U1026 or 1 µM JAK Inhibitor I. After 9 days, cells were trypsinized, counted and 3000 cells from each sample were inoculated on 6 cm dishes in the presence of irradiated 3T3 feeder cells. Cultures were grown for 2 weeks, when most colonies were 2–5 mm in diameter. After fixation and staining with Rhodanile blue^61^, colonies of approximately 50 cells or more were counted. Results were normalized to the untreated sample. The experiment was performed in triplicate.

## Supplementary information

Supplementary information.
