# Supplementary material for: Deducing signaling pathways from parallel actions of arsenite and antimonite in human epidermal keratinocytes
Source: Sci Rep. 2020 Feb 19;10:2890. doi: 10.1038/s41598-020-59577-0 (PMC7031270; doi:10.1038/s41598-020-59577-0)
Supplement: Supplementary file 1 — Supplementary information. [file 41598_2020_59577_MOESM1_ESM.pdf]

Deducing signaling pathways from parallel actions of arsenite and antimonite in human epidermal keratinocytes

Marjorie A. Phillips[1], Angela Cánovas[2], Miguel A. Rea[3], Alma Islas-Trejo[4], Juan F. Medrano[4], Blythe Durbin-Johnson[5], David M. Rocke[5], Robert H. Rice[1]

[1] Department of Environmental Toxicology, University of California, Davis, CA, USA

[2] Centre for Genetic Improvement of Livestock, Department of Animal Biosciences, University of Guelph, Guelph, ON N1G 2W1, Canada

[3] Department of Chemistry, Universidad Autónoma Querétaro, Mexico

[4] Department of Animal Science, University of California, Davis, CA, USA

[5] Division of Biostatistics, Department of Public Health Sciences, Clinical and Translational Science Center Biostatistics Core, University of California, Davis, CA, USA

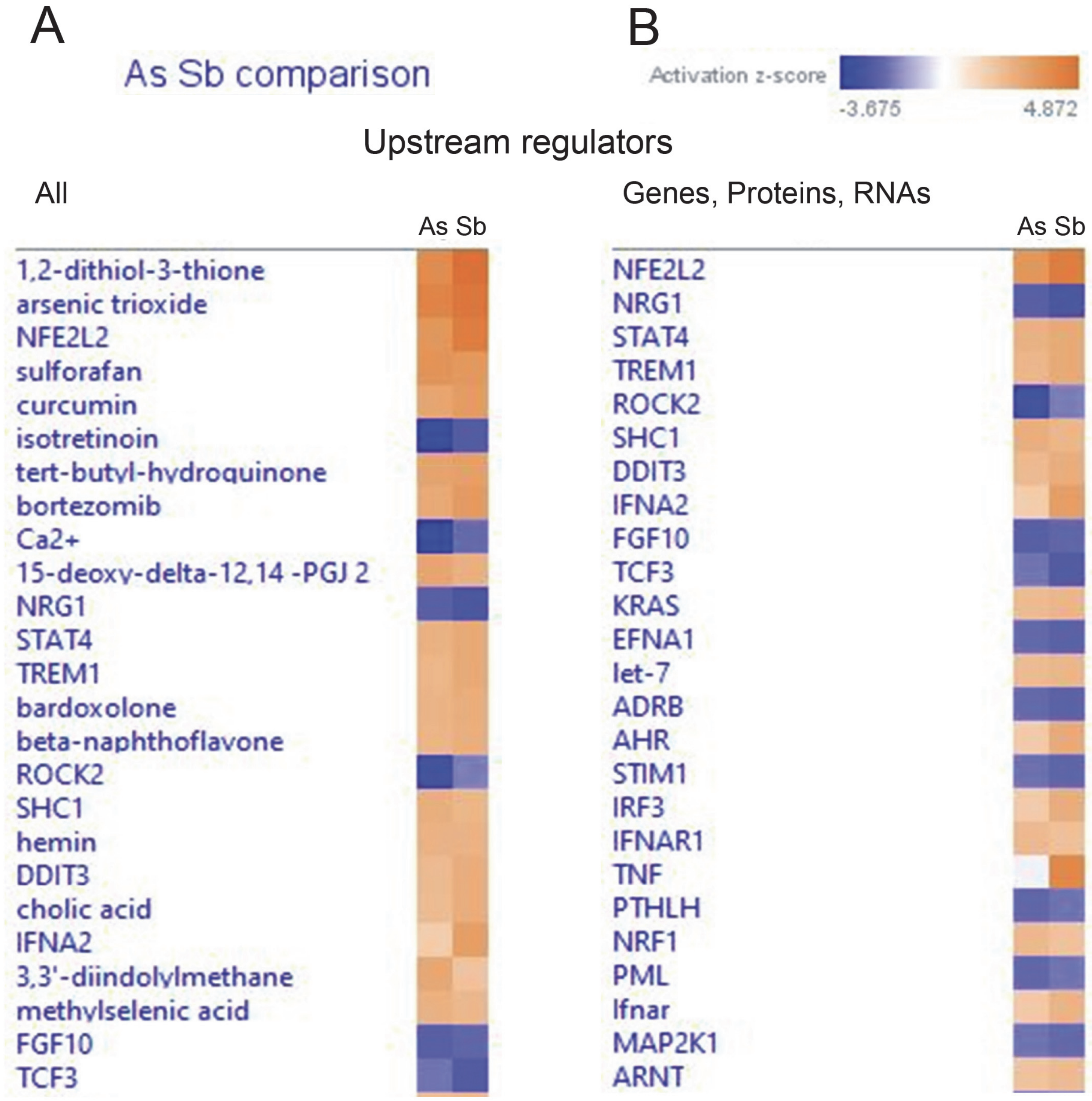

Supplementary Figure 1. Heat maps of predicted upstream regulators. IPA comparative analysis based on transcriptional changes after arsenite (As) and antimonite (Sb) treatments, sorted by z-score. (A) All predicted upstream regulators; (B) filtered for genes, proteins and RNAs.

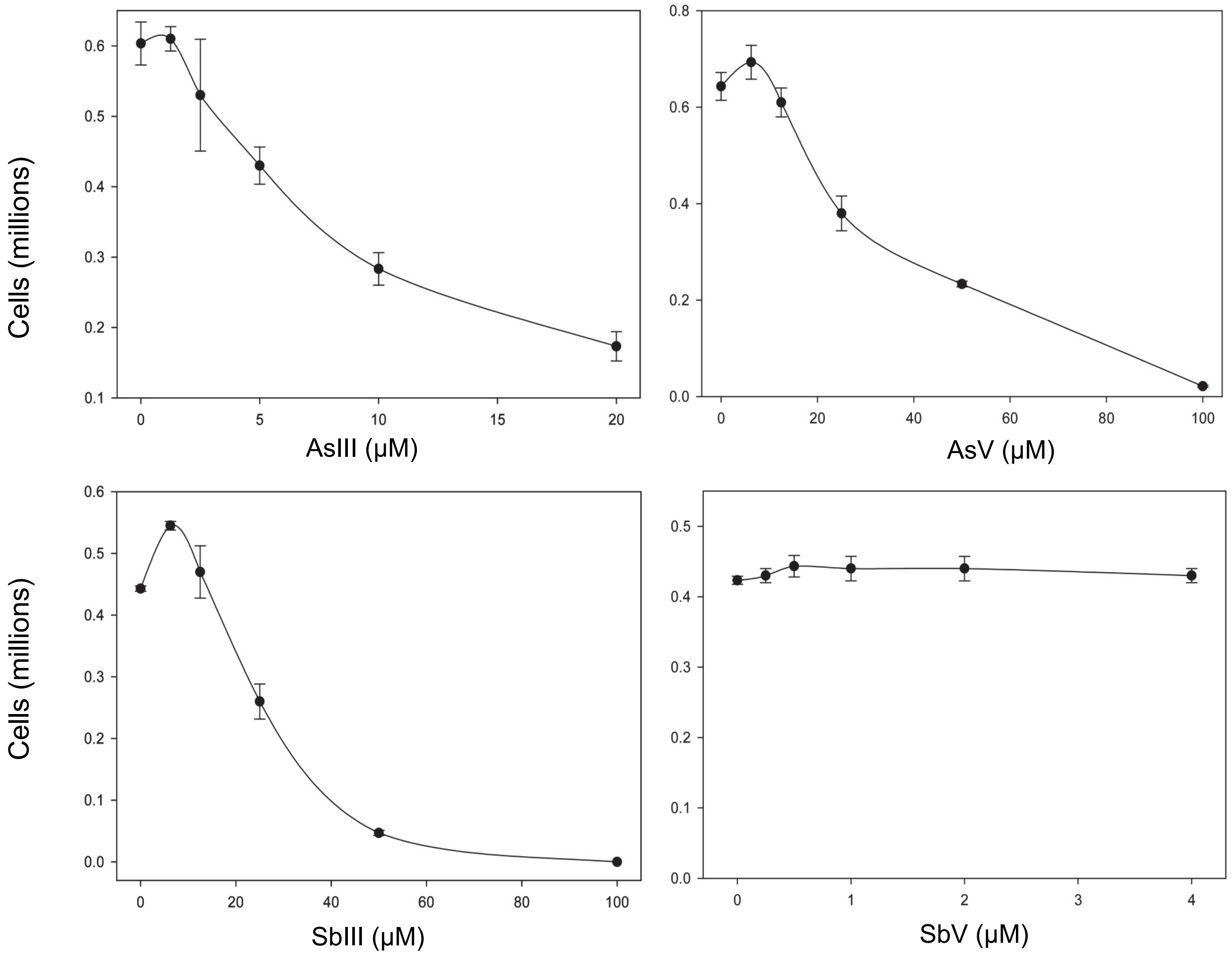

Supplementary Figure 2. Cell survival in SIK cultures treated with arsenite, arsenate, antimonite, antimonate. Triplicate cultures nearing confluence were treated at the indicated metalloid concentrations, and the remaining attached cells were counted after 4 days using a Beckman Coulter Multisizer3. EC^{50} values (µM) were estimated from the data as 9 (AsIII), 28 (SbIII), 28 (AsV) and >4000 (SbV).

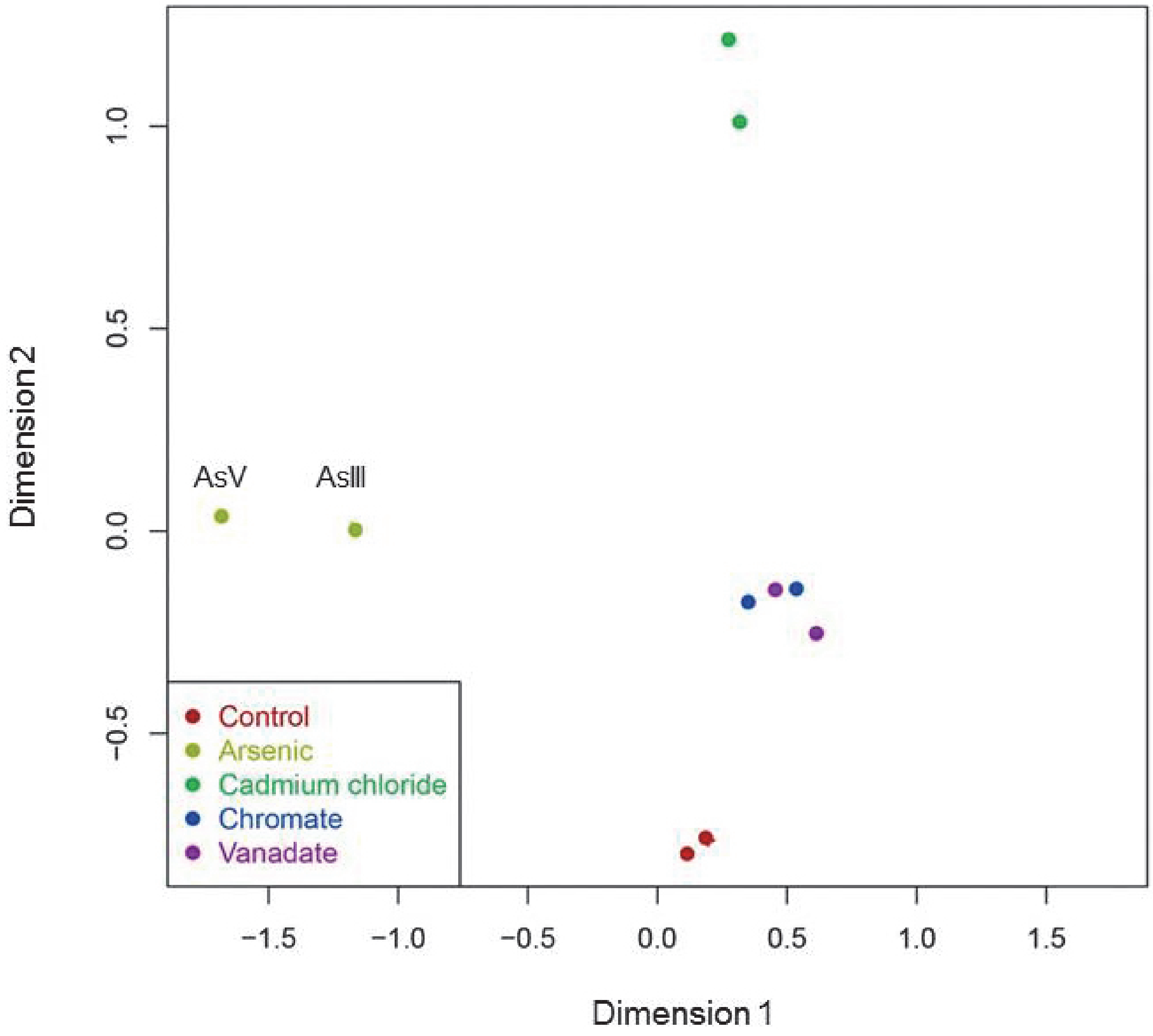

Supplementary Figure 3. Multidimensional scaling plot illustrating the distinct separation of responses to arsenic from those to other treatments (cadmium, chromate, vanadate).

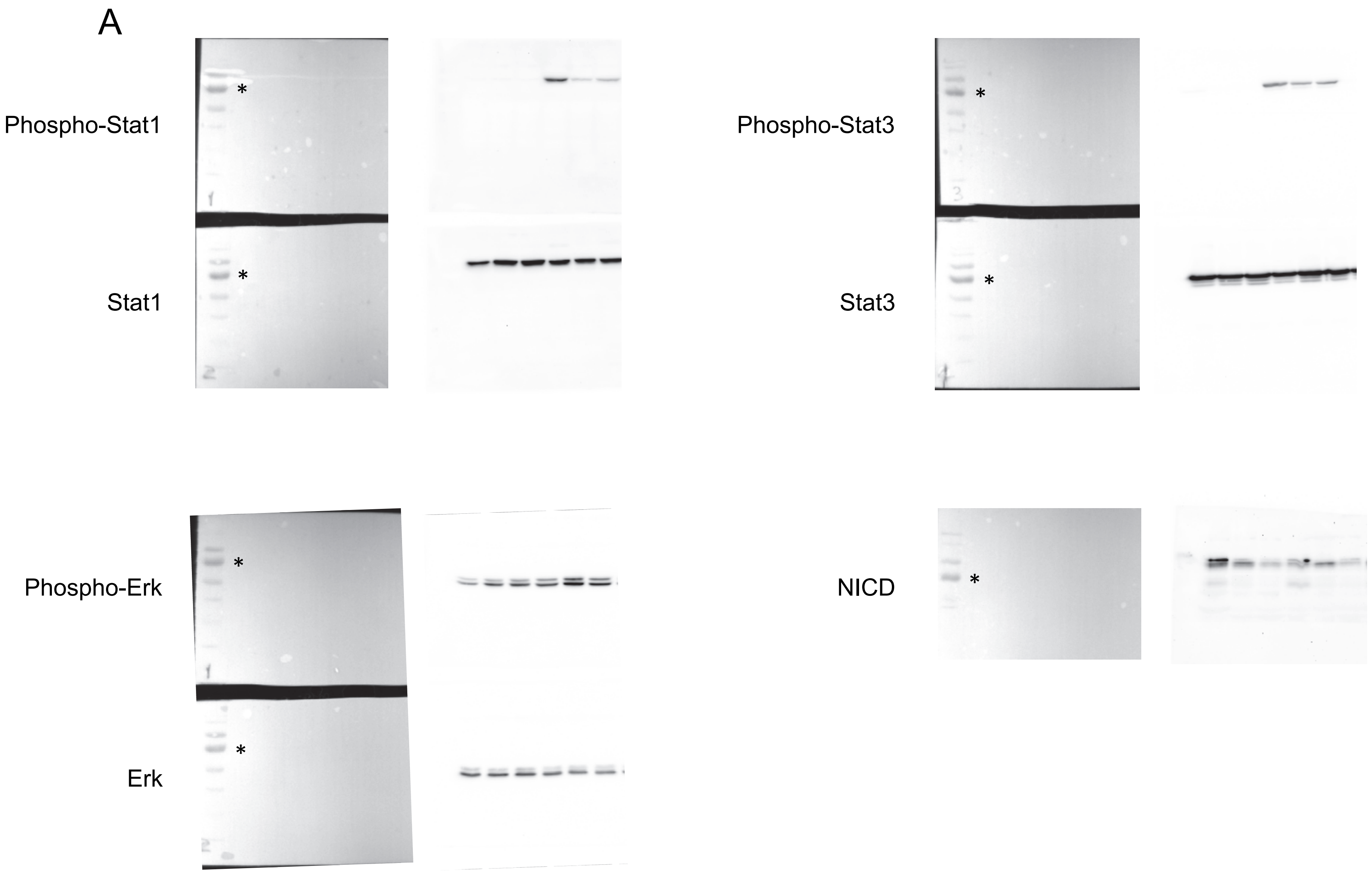
A
Phospho-Stat1
Stat1
Phospho-Stat3
Stat3
Phospho-Erk
Erk
NICD
*

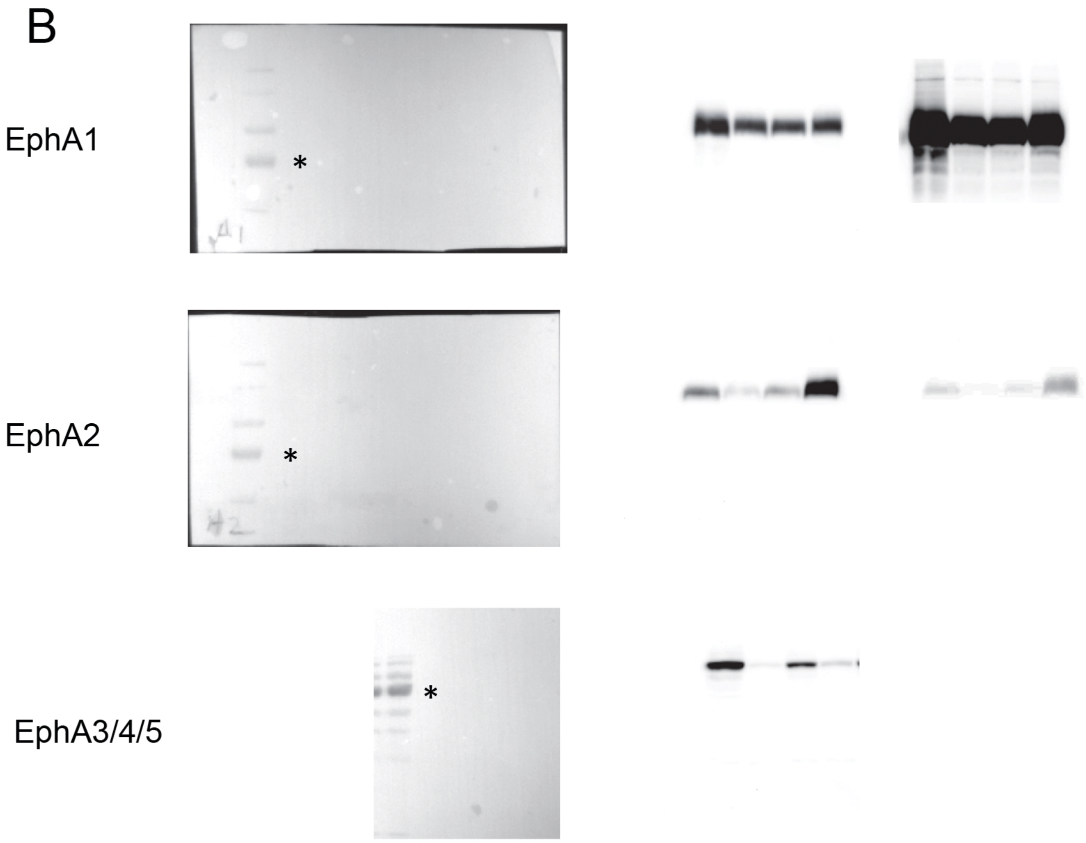

Supplementary Fig 4. Images of original western blots for Figures 4 (A) and (B). In each case, the image on the left shows PageRuler prestained molecular weight markers (250, 130, 95, 72 [indicated by asterisk], 55, 36 kDa), and the image on the right shows the corresponding antibody staining. For EphA1 and EphA2, alternate exposures of the blots are shown to the right of the images selected for Figure 4B.

Supplementary Table 1. Canonical Pathways. Yellow highlights indicate genes differentially expressed in parallel by As and Sb.

Nrf2 mediated oxidative stress

		As			Sb		
Symbol	Entrez Gene Name	Expr Fold Change	Expr False Discovery Rate (p-value)	Expr Intensity/RPKM/FPKM/Counts	Expr Fold Change	Expr False Discovery Rate (p-value)	Expr Intensity/RPKM/FPKM/Counts
ABCC2	ATP binding cassette subfamily C member 2	9.305	2.19E-19	52.677	12.205	1.04E-14	69.095
ACTG1	actin gamma 1	-2.565	0.0281	160.93	-1.287	1	320.723
CBR1	carbonyl reductase 1	2.855	2.65E-06	62.043	2.489	0.0037	54.089
DNAJB4	DnaJ heat shock protein family (Hsp40) member B4	2.187	0.00498	30.514	3.767	6.31E-07	52.563
EPHX1	epoxide hydrolase 1	5.307	1.57E-06	17.809	8.659	6.95E-12	29.058
FGFR3	fibroblast growth factor receptor 3	-2.068	0.0141	22.784	-6.483	2.36E-12	7.267
FOS	Fos proto-oncogene, AP-1 transcription factor subunit	-4.705	0.012	1.708	-4.595	0.00379	1.749
FTH1	ferritin heavy chain 1	6.341	1.49E-33	4816.884	5.732	3.51E-26	4353.958
FTL	ferritin light chain	10.144	2.09E-38	2637.527	13.967	4.71E-33	3631.653
GCLC	glutamate-cysteine ligase catalytic subunit	2.485	0.000104	148.936	2.264	1.48E-07	135.685
GCLM	glutamate-cysteine ligase modifier subunit	15.134	1.33E-18	29.883	30.081	7.11E-16	59.397
GPX2	glutathione peroxidase 2	8.94	0.000443	18.22	19.897	0.000648	40.551
HMOX1	heme oxygenase 1	80.801	1.23E-164	282.601	83.141	1.46E-38	290.784
HSPB8	heat shock protein family B (small) member 8	3.309	0.178	7.97	5.294	0.000264	12.752
MGST1	microsomal glutathione S-transferase 1	2.587	3.17E-13	308.953	2.966	2.97E-18	354.162
NQO1	NAD(P)H quinone dehydrogenase 1	13.853	4.47E-22	101.45	30.022	2.37E-29	219.855
PRDX1	peroxiredoxin 1	2.114	1.13E-10	356.786	2.894	3.91E-13	488.439
SOD1	superoxide dismutase 1	2.951	5.06E-16	196.387	2.615	2.37E-11	174.002
SQSTM1	sequestosome 1	4.567	5.63E-37	204.854	6.556	1.37E-14	294.105
TXN	thioredoxin	3.481	4.6E-11	348.059	7.638	2.3E-11	763.747
TXNRD1	thioredoxin reductase 1	8.831	3.29E-26	77.313	17.548	2.93E-30	153.634

Glucocorticoid Receptor Signaling

		As			Sb		
Symbol	Entrez Gene Name	Expr Fold Change	Expr False Discovery Rate (p-value)	Expr Intensity/RPKM/FPKM/Counts	Expr Fold Change	Expr False Discovery Rate (p-value)	Expr Intensity/RPKM/FPKM/Counts
CEBPA	CCAAT/enhancer binding protein alpha	-2.256	0.00229	11.77	-2.235	0.000858	11.876
FGFR3	fibroblast growth factor receptor 3	-2.068	0.0141	22.784	-6.483	2.36E-12	7.267
FOS	Fos proto-oncogene, AP-1 transcription factor subunit	-4.705	0.012	1.708	-4.595	0.00379	1.749
HLTF	helicase like transcription factor	3.499	0.0993	7.9	4.196	0.0171	9.475
HSPA8	heat shock protein family A (Hsp70) member 8	1.338	1	171.701	2.176	0.000033	279.298
HSPA1A/H:	heat shock protein family A (Hsp70) member 1A	1.495	0.46	50.133	2.113	0.000116	70.851
IL1RN	interleukin 1 receptor antagonist	-4.209	1.24E-20	15.712	-2.749	3.01E-11	24.058
MAP2K1	mitogen-activated protein kinase kinase 1	1.675	0.624	26.867	2.097	0.0127	33.649
PBX1	PBX homeobox 1	-3.581	0.106	1.973	-4.886	0.00713	1.446
PIK3R1	phosphoinositide-3-kinase regulatory subunit 1	-1.94	0.135	9.474	-2.98	0.000269	6.167
PIK3R2	phosphoinositide-3-kinase regulatory subunit 2	-2.322	0.242	4.786	-3.037	0.0137	3.659
POLR2J2/P	RNA polymerase II subunit J2	-2.079	0.00801	18.501	-2.585	0.000288	14.877
SERPINE1	serpin family E member 1	3.447	8.62E-06	98.901	3.555	0.00471	102.018
STAT1	signal transducer and activator of transcription 1	1.716	0.944	18.206	2.846	0.0000982	30.2
TSC22D3	TSC22 domain family member 3	-1.108	1	35.908	-3.595	0.000278	11.072

Retinoate Biosynthesis

		As			Sb		
Symbol	Entrez Gene Name	Expr Fold Change	Expr False Discovery Rate (p-value)	Expr Intensity/RPKM/FPKM/Counts	Expr Fold Change	Expr False Discovery Rate (p-value)	Expr Intensity/RPKM/FPKM/Counts
AKR1B10	aldo-keto reductase family 1 member B10	12.173	9.86E-12	52.745	26.567	1.13E-14	115.114
AKR1C3	aldo-keto reductase family 1 member C3	20.66	6.27E-23	78.06	40.652	6.34E-23	153.597
DHRS9	dehydrogenase/reductase 9	-32.596	0.000615	0.251	-6.191	0.0265	1.323
RDH12	retinol dehydrogenase 12 (all-trans/9-cis/11-cis)	-13.018	0.0697	0.642	1.147	1	9.59
SDR16C5	short chain dehydrogenase/reductase family 16C member 5	-3.982	0.0306	1.89	-1.269	1	5.93
SDR9C7	short chain dehydrogenase/reductase family 9C member 7	-9.745	0.0171	0.754	-2.093	1	3.509

Xenobiotic Metabolism Signaling

Symbol	Entrez Gene Name	As Expr Fold Change	As Expr False Discovery Rate (p-value)	As Expr Intensity/RPKM/FPKM/Counts	Sb Expr Fold Change	Sb Expr False Discovery Rate (p-value)	Sb Expr Intensity/RPKM/FPKM/Counts
ABCC2	ATP binding cassette subfamily C member 2	9.305	2.19E-19	52.677	12.205	1.04E-14	69.095
ABCC3	ATP binding cassette subfamily C member 3	1.222	1	1.699	7.398	0.00106	10.287
AIP	aryl hydrocarbon receptor interacting protein	1.786	0.555	21	2.108	0.0618	24.78
ALDH3A1	aldehyde dehydrogenase 3 family member A1	2.476	0.265	10.954	2.768	0.121	12.25
ALDH3A2	aldehyde dehydrogenase 3 family member A2	2.309	2.21E-09	99.708	2.731	4.93E-13	117.913
ALDH3B2	aldehyde dehydrogenase 3 family member B2	-7.74	1.43E-09	4.161	-2.943	0.00483	10.945
CYP1B1	cytochrome P450 family 1 subfamily B member 1	-12.938	5.18E-07	0.864	-15.317	2.33E-07	0.73
FGFR3	fibroblast growth factor receptor 3	-2.068	0.0141	22.784	-6.483	2.36E-12	7.267
FTL	ferritin light chain	10.144	2.09E-38	2637.527	13.967	4.71E-33	3631.653
GCLC	glutamate-cysteine ligase catalytic subunit	2.485	0.000104	148.936	2.264	1.48E-07	135.685
GSTO1	glutathione S-transferase omega 1	1.634	0.431	38.089	2.363	0.000286	55.089
HMOX1	heme oxygenase 1	80.801	1.23E-164	282.601	83.141	1.46E-38	290.784
MAOA	monoamine oxidase A	-1.819	0.347	17.642	-2.988	0.00193	10.74
MAP2K1	mitogen-activated protein kinase kinase 1	1.675	0.624	26.867	2.097	0.0127	33.649
MGST1	microsomal glutathione S-transferase 1	2.587	3.17E-13	308.953	2.966	2.97E-18	354.162
NFKB2	nuclear factor kappa B subunit 2	2.177	0.372	13.581	3.259	0.000456	20.331
NQO1	NAD(P)H quinone dehydrogenase 1	13.853	4.47E-22	101.45	30.022	2.37E-29	219.855
NQO2	N-ribosyldihydronicotinamide:quinone reductase 2	4.026	0.327	5.667	4.543	0.082	6.394
PIK3R1	phosphoinositide-3-kinase regulatory subunit 1	-1.94	0.135	9.474	-2.98	0.000269	6.167
PIK3R2	phosphoinositide-3-kinase regulatory subunit 2	-2.322	0.242	4.786	-3.037	0.0137	3.659
PPP2R2C	protein phosphatase 2 regulatory subunit Bgamma	-5.981	0.0000304	2.067	-2.188	0.115	5.651
SULT1C3	sulfotransferase family 1C member 3	-11.601	0.000134	0.696	-3.547	0.0359	2.277
SULT1E1	sulfotransferase family 1E member 1	-2.1	0.0708	13.684	-3.593	6.51E-06	7.999
SULT2B1	sulfotransferase family 2B member 1	-5.421	2.11E-07	5.693	-2.384	0.0109	12.944
UGT1A6	UDP glucuronosyltransferase family 1 member A6	6.624	6.57E-28	104.796	6.154	2.23E-24	97.36

γ-glutamyl cycle

Symbol	Entrez Gene Name	As Expr Fold Change	As Expr False Discovery Rate (p-value)	As Expr Intensity/RPKM/FPKM/Counts	Sb Expr Fold Change	Sb Expr False Discovery Rate (p-value)	Sb Expr Intensity/RPKM/FPKM/Counts
GCLC	glutamate-cysteine ligase catalytic subunit	2.485	0.000104	148.936	2.264	1.48E-07	135.685
GCLM	glutamate-cysteine ligase modifier subunit	15.134	1.33E-18	29.883	30.081	7.11E-16	59.397
GGT1	gamma-glutamyltransferase 1	5.733	0.052	6.328	6.589	0.0111	7.272
GGT6	gamma-glutamyltransferase 6	-2.305	0.0574	6.534	-1.22	1	12.344

Vitamin C transport

Symbol	Entrez Gene Name	As Expr Fold Change	As Expr False Discovery Rate (p-value)	As Expr Intensity/RPKM/FPKM/Counts	Sb Expr Fold Change	Sb Expr False Discovery Rate (p-value)	Sb Expr Intensity/RPKM/FPKM/Counts
GLRX	glutaredoxin	6.523	0.00408	7.938	15.831	1.09E-10	19.264
GSTO1	glutathione S-transferase omega 1	1.634	0.431	38.089	2.363	0.000286	55.089
SLC2A3	solute carrier family 2 member 3	3.279	0.12	14.237	4.388	0.00302	19.049
TXN	thioredoxin	3.481	4.6E-11	348.059	7.638	2.3E-11	763.747
TXNRD1	thioredoxin reductase 1	8.831	3.29E-26	77.313	17.548	2.93E-30	153.634

Supplementary Table 2. Predicted Regulators for Arsenite

Upstream Regulator	Expr Fold Change	Molecule Type	Predicted Activation State	Activ-ation z-score	p-value of overlap
arsenic trioxide		chemical drug	Activated	4.054	2.87E-17
1,2-dithiol-3-thione		chemical reagent	Activated	3.809	2.59E-10
sulforafan		chemical drug	Activated	3.484	6.87E-13
NFE2L2	-1.506	transcription regulator	Activated	3.385	1.61E-18
tert-butyl-hydroquinone		chemical reagent	Activated	2.938	9.3E-10
curcumin		chemical drug	Activated	2.876	2.14E-06
15-deoxy-delta-12,14 -PGJ 2		chemical - endogenous non-mammalian	Activated	2.856	8.17E-05
3,3'-diindolylmethane		chemical drug	Activated	2.778	1.77E-05
GATA3	-1.509	transcription regulator	Activated	2.742	2.66E-05
bortezomib		chemical drug	Activated	2.701	0.00238
SHC1	-1.373	other	Activated	2.56	6.92E-05
INSIG1	1.035	other	Activated	2.522	4.06E-05
STAT4	2.819	transcription regulator	Activated	2.46	0.0054
methylselenic acid		chemical reagent	Activated	2.449	0.00531
xanthohumol		chemical - endogenous non-mammalian	Activated	2.414	5.89E-07
hemin		chemical - endogenous mammalian	Activated	2.405	0.00118
bardoxolone		chemical drug	Activated	2.376	1.69E-08
beta-naphthoflavone		chemical toxicant	Activated	2.362	3.61E-09
TREM1	-3.273	transmembrane receptor	Activated	2.319	0.00235
fluocinolone acetonide		chemical drug	Activated	2.236	0.000486
GRB2	1.09	kinase	Activated	2.236	0.00248
SOX7	-2.498	transcription regulator	Activated	2.236	0.00134
PAX1	1	transcription regulator	Activated	2.236	0.00226
caffeic acid phenethyl ester		chemical drug	Activated	2.229	0.00413
NRF1	1.007	transcription regulator	Activated	2.219	0.000388
DDIT3	1.532	transcription regulator	Activated	2.191	0.0062
IFNAR1	1.333	transmembrane receptor	Activated	2.19	0.0152
ATF4	1.181	transcription regulator	Activated	2.19	0.0835
sodium arsenite		chemical drug	Activated	2.181	1.2E-06
S-adenosylmethionine		chemical - endogenous mammalian	Activated	2.18	0.000484
let-7	1	microrna	Activated	2.177	0.166
cholic acid		chemical - endogenous mammalian	Activated	2.173	0.0177
triamcinolone acetonide		chemical drug	Activated	2.173	0.00119
IgG		complex	Activated	2.169	1.85E-10
KRAS	-1.134	enzyme	Activated	2.164	4.42E-08
Immunoglobulin		complex	Activated	2.142	0.0011
nitric oxide		chemical - endogenous mammalian	Activated	2.078	1.47E-07
paraquat		chemical toxicant	Activated	2.058	0.00219
Alpha catenin		group	Activated	2.022	2.93E-05
motexafin gadolinium		chemical drug	Activated	2	1.07E-05
PLIN5	1	other	Activated	2	0.00227
N-propyl bromide		chemical reagent	Activated	2	1.07E-05
ITK	1	kinase	Activated	2	0.081
diquat		chemical toxicant	Activated	2	5.43E-06
pentoxifylline		chemical drug	Activated	2	0.00576

STIM1		ion channel	Inhibited	-2	0.000186
F7	-3.313	peptidase	Inhibited	-2	0.00374
GLIS1	-2.407	transcription regulator	Inhibited	-2	1.89E-05
KLK5	1.577	peptidase	Inhibited	-2	0.000398
glutathione		chemical - endogenous mammalian	Inhibited	-2.005	0.000127
CSF1	-4.659	cytokine	Inhibited	-2.008	0.0222
GLI1	-5.808	transcription regulator	Inhibited	-2.022	0.115
trinitrobenzenesulfonic acid		chemical reagent	Inhibited	-2.063	0.00243
PRKCD	-1.458	kinase	Inhibited	-2.065	0.00311
SRC	-1.261	kinase	Inhibited	-2.109	0.000121
ADRB		group	Inhibited	-2.111	0.000129
SOX2	1	transcription regulator	Inhibited	-2.121	0.000662
PDX1	1	transcription regulator	Inhibited	-2.121	0.00925
PTGS2	-4.815	enzyme	Inhibited	-2.129	0.0172
EFNA1	1.139	other	Inhibited	-2.138	5.93E-06
CRH	1	cytokine	Inhibited	-2.143	0.00766
cocaine		chemical drug	Inhibited	-2.146	0.267
CLDN7	-3.514	other	Inhibited	-2.164	3.99E-05
KLF4	-1.631	transcription regulator	Inhibited	-2.166	8.46E-07
EHF	-1.771	transcription regulator	Inhibited	-2.176	1.79E-12
PTGER2	-16.838	g-protein coupled receptor	Inhibited	-2.183	0.0613
PML	1.337	transcription regulator	Inhibited	-2.193	3.45E-08
PTHLH	-1.243	other	Inhibited	-2.194	0.000516
calcitriol		chemical drug	Inhibited	-2.325	1.56E-05
S100A8	-25.485	other	Inhibited	-2.345	0.00299
S100A9	-30.569	other	Inhibited	-2.351	0.00186
mibolerone		chemical drug	Inhibited	-2.414	0.00639
NRG1	-2.121	growth factor	Inhibited	-2.415	0.000602
FGF10	-25.673	growth factor	Inhibited	-2.423	0.000667
phorbol myristate acetate		chemical drug	Inhibited	-2.539	4.7E-06
ROCK2	-1.131	kinase	Inhibited	-3.058	4.76E-14
isotretinoin		biologic drug	Inhibited	-3.503	1.29E-12
Ca2+		chemical - endogenous mammalian	Inhibited	-3.675	7.03E-08

Supplementary Table 3. Predicted regulators for antimonite. Results from Ingenuity Pathway Anlaysis.

Upstream Regulator	Expr Fold Change	Molecule Type	Predicted Activation State	Activ-ation z-score	p-value of overlap
1,2-dithiol-3-thione		chemical reagent	Activated	4.872	6.8E-11
arsenic trioxide		chemical drug	Activated	4.604	4E-12
NFE2L2	-1.25	transcription regulator	Activated	4.319	1.6E-18
TNF	1.024	cytokine	Activated	3.935	6E-20
sulforafan		chemical drug	Activated	3.341	1.5E-09
bortezomib		chemical drug	Activated	3.256	9.7E-05
curcumin		chemical drug	Activated	3.204	0.0005
IFNL1	1	cytokine	Activated	3.162	1.7E-05
IFNA2	1	cytokine	Activated	3.112	3.6E-09
tert-butyl-hydroquinone		chemical reagent	Activated	3.073	2.9E-08
IFNG	1	cytokine	Activated	2.816	4.4E-11
IFN Beta		group	Activated	2.808	7.4E-05
STAT4	4.04	transcription regulator	Activated	2.698	0.0002
HIF1A	-1.036	transcription regulator	Activated	2.697	4.3E-06
TREM1	-1.052	transmembrane receptor	Activated	2.683	3.8E-06
IFNB1	10.3	cytokine	Activated	2.666	0.00028
AHR	-1.217	ligand-dependent nuclear receptor	Activated	2.659	4.5E-06
Interferon alpha		group	Activated	2.644	0.00066
cobalt chloride		chemical reagent	Activated	2.6	0.00743
bromodeoxyuridine		chemical drug	Activated	2.598	0.00119
tunicamycin		chemical - endogenous non-mammalian	Activated	2.587	0.234
DDIT3	2.022	transcription regulator	Activated	2.584	0.00204
cholic acid		chemical - endogenous mammalian	Activated	2.581	0.00859
IRF3	1.171	transcription regulator	Activated	2.564	0.00094
bardoxolone		chemical drug	Activated	2.563	5.2E-08
TLR9	-2.011	transmembrane receptor	Activated	2.561	0.00686
poly rI:rC-RNA		biologic drug	Activated	2.546	6.8E-05
beta-naphthoflavone		chemical toxicant	Activated	2.538	1.7E-11
E. coli serotype 0127B8 lipopolysaccharide		chemical - endogenous non-mammalian	Activated	2.496	0.00546
15-deoxy-delta-12,14 -PGJ 2		chemical - endogenous non-mammalian	Activated	2.496	5.1E-05
OSM	1	cytokine	Activated	2.464	4E-22
enterotoxin B		biologic drug	Activated	2.449	0.0242
motexafin gadolinium		chemical drug	Activated	2.449	2.3E-08
chenodeoxycholic acid		chemical - endogenous mammalian	Activated	2.413	0.0146
hemin		chemical - endogenous mammalian	Activated	2.405	0.00045
genistein		chemical drug	Activated	2.387	0.00011
Ifnar		group	Activated	2.378	0.00138
IFNA1/IFNA13	1.74	cytokine	Activated	2.375	0.00636
IRF7	2.228	transcription regulator	Activated	2.371	0.0679
KRAS	1.036	enzyme	Activated	2.325	3.4E-11
pirinixic acid		chemical toxicant	Activated	2.323	7.1E-06
SHC1	-1.568	other	Activated	2.305	2.4E-05
let-7	1.359	microrna	Activated	2.286	0.0357
paraquat		chemical toxicant	Activated	2.274	0.0091
indomethacin		chemical drug	Activated	2.259	0.0175
TLR3	1.229	transmembrane receptor	Activated	2.249	0.00153
JAK1	1.049	kinase	Activated	2.236	0.0124
niacinamide		chemical - endogenous mammalian	Activated	2.236	0.0124
methylselenic acid		chemical reagent	Activated	2.236	0.0125
flutamide		chemical drug	Activated	2.236	2.6E-05
prostaglandin J2		chemical - endogenous non-mammalian	Activated	2.213	0.00246

deoxycholate		chemical - endogenous mammalian	Activated	2.213	0.0205
FOXO3	1.025	transcription regulator	Activated	2.21	1.9E-05
stallimycin		biologic drug	Activated	2.186	0.0136
sodium arsenite		chemical drug	Activated	2.181	3.8E-05
CpG ODN 1826		chemical reagent	Activated	2.176	0.0359
10E,12Z-octadecadienoic acid		chemical - endogenous mammalian	Activated	2.171	0.00013
TLR7	1	transmembrane receptor	Activated	2.158	0.439
DDX58	3.774	enzyme	Activated	2.157	0.0336
cadmium		chemical toxicant	Activated	2.157	0.0797
cephaloridine		chemical drug	Activated	2.141	1.3E-05
ARNT	1.46	transcription regulator	Activated	2.132	3.1E-06
aldesleukin		biologic drug	Activated	2.121	8.9E-05
deferoxamine		chemical drug	Activated	2.091	3.6E-06
gentamicin		chemical drug	Activated	2.089	0.00427
IRF1	1.132	transcription regulator	Activated	2.085	0.00428
TNFSF10	-1.541	cytokine	Activated	2.084	9.3E-06
acetaminophen		chemical drug	Activated	2.07	0.018
decitabine		chemical drug	Activated	2.067	8E-17
S-adenosylmethionine		chemical - endogenous mammalian	Activated	2.038	5.7E-06
CDKN2A	1.162	transcription regulator	Activated	2.017	0.059
EPAS1	-1.206	transcription regulator	Activated	2.011	0.00045
IL15	3.35	cytokine	Activated	2.007	0.111
EPO	-1.695	cytokine	Activated	2.007	0.0178
nitrofen		chemical toxicant	Activated	2	0.0294
PLIN5	3.949	other	Activated	2	0.0104
N-propyl bromide		chemical reagent	Activated	2	5.8E-05
MAFF	1.577	transcription regulator	Activated	2	0.00017
NFYA	1.414	transcription regulator	Activated	2	0.0137
IFNE	-1.167	cytokine	Activated	2	0.0246
DPP-23		chemical reagent	Activated	2	0.00245
reactive oxygen species		chemical toxicant	Activated	2	0.174
diquat		chemical toxicant	Activated	2	3E-05
pentoxifylline		chemical drug	Activated	2	0.0246
6-hydroxydopamine		chemical toxicant	Activated	2	0.115
arsenic		chemical toxicant		1.993	0.0013
IFN type 1		group		1.987	0.0611
TRAP1	-1.036	enzyme	Inhibited	-2	0.204
CBFB	1.314	transcription regulator	Inhibited	-2	0.197
BNIP3L	2.566	other	Inhibited	-2	0.0908
MAP3K8	-1.551	kinase	Inhibited	-2	0.487
Ca2+		chemical - endogenous mammalian	Inhibited	-2.109	6E-05
EFNA3	-1.607	kinase	Inhibited	-2.111	5.5E-08
MITF	1.784	transcription regulator	Inhibited	-2.121	0.455
CD44	1.085	other	Inhibited	-2.126	0.00135
MAP2K1	2.097	kinase	Inhibited	-2.126	0.00034
miR-133a-3p/other miRNAs w/seed UUGGUCC		mature microrna	Inhibited	-2.176	0.0205
Growth hormone		group	Inhibited	-2.18	0.00203
prednisolone		chemical drug	Inhibited	-2.183	0.0008
PTH	1	other	Inhibited	-2.213	0.194
FGF10	-13.792	growth factor	Inhibited	-2.219	0.0239
RTN4	1.707	other	Inhibited	-2.219	0.00528
miR-30c-5p/other miRNAs w/seed GUAAACA		mature microrna	Inhibited	-2.236	0.25
STIM1		ion channel	Inhibited	-2.236	6.6E-05
COMMD1	1.316	transporter	Inhibited	-2.236	7.3E-05
FGFR2	-1.491	kinase	Inhibited	-2.241	0.0167

Vegf		group	Inhibited	-2.295	0.0913
ADRB		group	Inhibited	-2.324	3.1E-05
EFNA4	1.016	kinase	Inhibited	-2.331	3.4E-09
EFNA1	1.147	other	Inhibited	-2.345	0.00017
glutathione		chemical - endogenous mammalian	Inhibited	-2.418	0.00759
isotretinoin		biologic drug	Inhibited	-2.554	4E-07
miR-16-5p/other miRNAs w/seed AGCAGCA		mature microrna	Inhibited	-2.557	0.011
TCF3	-1.011	transcription regulator	Inhibited	-2.577	0.151
NRG1	-1.226	growth factor	Inhibited	-2.775	0.0016
salirasib		chemical drug	Inhibited	-2.813	0.00019
NR3C1	1.153	ligand-dependent nuclear receptor	Inhibited	-2.816	1.5E-07

Supplementary Table 4. Effects of Erk and Stat pathway inhibitors on gene expression changes induced by arsenite, antimonite and OSM.

Treatment	*KRT1*	*KRT10*	*DSG1*	*DSC1*	*FLG*
As	0.06±0.004	0.02±0.002	0.09±0.002	0.003±0.0004	0.03±0.001
As+U1026	0.5±0.1	0.4±0.07	0.6±0.09	0.2±0.07	0.2±0.002
As+Jak inhibitor	0.001±0.00007	0.001±0.0003	0.008±0.001	0.004±0.0006	0.02±0.002
Sb	0.1±0.002	0.03±0.003	0.06±0.005	0.005±0.0004	0.1±0.01
Sb+U1026	2.1±0.2	0.9±0.1	0.9±0.004	1.2±0.1	2.7±0.2
Sb+Jak inhibitor	0.001±0.0002	0.001±0.0001	0.007±0.0004	0.004±0.0007	0.03±0.007
OSM	0.004±0.0005	0.005±0.0006	0.03±0.002	0.009±0.0009	0.01±0.001
OSM+U1026	0.01±0.002	0.005±0.0001	0.01±0.002	0.002±0.0002	0.008±0.0001
OSM+Jak inhibitor	0.2±0.04	0.4±0.05	0.4±0.03	0.1±0.02	0.1±0.02
OSM+U1026+Jak inhibitor	1.3±0.3	1.2±0.2	1.7±0.1	2.1±0.2	4.6±0.7

mRNA levels: ratios compared to levels in untreated cells. Numbers are the averages and standard deviations of triplicate samples. U1026 is an inhibitor of MEK.

Supplementary Table 5. Transcription factor genes differentially expressed as a result of treatment by arsenite and antimonite.

Gene ID	Gene Name	Fold Change	
		**As**	**Sb**
SQSTM1	sequestosome 1	4.6	6.5
HTATIP2	HIV-1 Tat interactive protein 2	2.9	3.3
NUPR1	nuclear protein 1, transcriptional regulator	2.4	2.6*
PIR	pirin	2.2	2.4
ID3	inhibitor of DNA binding 3, HLH protein	2.1	1.6*
CALR	calreticulin	1.8	2.3
CNBP	CCHC-type zinc finger nucleic acid binding protein	1.8	2.2
BTF3	basic transcription factor 3	-1.3	-1.6
IRF6	interferon regulatory factor 6	-1.4	-1.3*
ID1	inhibitor of DNA binding 1, HLH protein	-1.5	-1.5
HMGA1	high mobility group AT-hook 1	-1.6	-1.2*
EHF	ETS homologous factor	-1.8	-1.6
BTG2	BTG anti-proliferation factor 2	-1.8	-2.4
TP63	tumor protein p63	-1.9	-2
DNAJB1	DnaJ heat shock protein family (Hsp40) member B1-1.9	-2	
POLR2J2/3	RNA polymerase II subunit J2	-2.1	-2.6
KLF5	Kruppel like factor 5	-2.1	-1.9
CEBPA	CCAAT/enhancer binding protein alpha	-2.3	-2.2
BHLHE40	basic helix-loop-helix family member e40	-2.3	-1.1*
BARX2	BARX homeobox 2	-2.5	-2.4
CDKN2B	cyclin dependent kinase inhibitor 2B	-2.6	-2.9
FOXQ1	forkhead box Q1	-2.8	-7.9
IKZF2	IKAROS family zinc finger 2	-3.0	-3.3
HOPX	HOP homeobox	-4.5	-5.4
FOS	Fos proto-oncogene, AP-1 transcription factor subunit	-4.7	-4.6
GRHL1	grainyhead like transcription factor 1	-6.1	-2.6
ELF3	E74 like ETS transcription factor 3	-10.5	-7.6
PITX1	paired like homeodomain 1	-12.1	-16
ANKRD22	ankyrin repeat domain 22	-32.6	-3.3*
EGR3	early growth response 3	-32.6	-17.6

Transcription factor genes differentially expressed after treatment with arsenite at $p \leq 0.05$. Genes were also differentially expressed by treatment with antimonite. $p \leq 0.05$ except *.
